# Effects of simultaneous pancreas-kidney transplantation and kidney transplantation alone on the outcome of peripheral vascular diseases

**DOI:** 10.1186/s12882-019-1649-7

**Published:** 2019-12-09

**Authors:** Robert Sucher, Sebastian Rademacher, Nora Jahn, Max Brunotte, Tristan Wagner, Athanasios Alvanos, Elisabeth Sucher, Daniel Seehofer, Uwe Scheuermann, Hans-Michael Hau

**Affiliations:** 1 Department of Visceral, Transplantation, Vascular and Thoracic Surgery, University Hospital of Leipzig, Leipzig, Germany; 2 Department of Anesthesiology and Intensive Care Medicine, University Hospital of Leipzig, Leipzig, Germany; 3 Department of Gastroenterology, University Hospital of Leipzig, Leipzig, Germany; 4 Department of Surgery, University Hospital of Leipzig, Liebigstrasse 20, 04103, Leipzig, Germany

**Keywords:** Pancreas transplantation, Kidney transplantation alone, Diabetes mellitus, Peripheral arterial disease, Diabetic vasculopathy, Vascular steal syndrome

## Abstract

**Background:**

The effects of Simultaneous Pancreas Kidney Transplantation (SPKT) on Peripheral Vascular Disease (PVD) warrants additional study and more target focus, since little is known about the mid- and long-term effects on the progression of PVD after transplantation.

**Methods:**

101 SPKT and 26 Kidney Transplantation Alone (KTA) recipients with insulin-dependent diabetes mellitus (IDDM) were retrospectively evaluated with regard to graft and metabolic outcome. Special subgroup analysis was directed towards the development and progression of peripheral vascular complications (PVC) (amputation, ischemic ulceration, lower extremity angioplasty/ bypass surgery) after transplantation.

**Results:**

The 10-year patient survival was significantly higher in the SPKT group (SPKT: 82% versus KTA 40%; *P* < 0.001). KTA recipients had a higher prevalence of atherosclerotic risk factors, including coronary artery disease (*P* < 0.001), higher serum triglyceride levels (*P* = 0.049), higher systolic (*P* = 0.03) and diastolic (*P* = 0.02) blood pressure levels. The incidence of PVD before transplantation was comparable between both groups (*P* = 0.114). Risk factor adjusted multivariate analysis revealed that patients with SPKT had a significant lower amount (32%) of PVCs (32 PVCs in 21 out of 101 SPKT; *P* < 0.001) when compared to the KTA patients who developed a significant increase in PVCs to 69% of cases (18 PVCs in 11 out of 26 KTA; *P* < 0.001). In line mean values of HbA_1c_ (*P* < 0.01) and serum triglycerides (*P* < 0.01) were significantly lower in patients with SPKT > 8 years after transplantation.

**Conclusion:**

SPKT favorably slows down development and progression of PVD by maintaining a superior metabolic vascular risk profile in patients with IDDM1.

## Introduction

Simultaneous transplantation of the pancreas and kidney (SPKT) still represents the gold standard surgical treatment for patients with insulin dependent diabetes mellitus type-1 (IDDM1) and end stage renal disease (ESRD) [1]. Diabetes mellitus is one of the main risk factors for the development and progression of peripheral vascular disease (PVD), which together with other macrovascular syndromes such as stroke and myocardial infarction represent one of the major causes of death in SPKT patients with functioning grafts [2]. However, successful pancreas transplantation leads to euglycemia, which could slow the progression of diabetic micro- and macrovascular complications [3].

In this context, it has been reported that long-term normoglycemia following successful SPKT sustains significant improvement of diabetic retinopathy and glomerulosclerosis, and that the human kidney even has the potential to substantially restore glomerular and tubular structures that have been previously injured by long term diabetes [3–8].

In contrast, data on the potential reversal or progression of macroangiopathic lesions by SPKT are far from clear. On one hand several studies report a significant higher incidence of PVD and worse vascular outcomes in patients with a SPKT when compared to those receiving a kidney transplant alone (KTA) [9]. A recent long-term study on incidence and risk factors in SPKT patients also reported a higher incidence of amputation after SPKT (up to 8.64%) when compared to KTA [10].

On the other hand, there is a big amount of data implying that progression of macrovascular disease is reduced in patients with IDDM1 after more than 5 years of successful combined pancreas-kidney transplantation when compared to kidney transplantation alone [11, 12]. A plethora of conflicting reports lead to the conclusion that the effects of SPKT on macrovascular disease are far from clear and warrant additional study and more target focus.

Therefore, the aim of this study was to determine the impact of SPKT and KTA on the progression of PAD and the development of lower-extremity ischemia, in a group of patients with diabetic vasculopathy and to some extent preexisting peripheral arterial disease (PAD). Additionally, the vascular risk factors and metabolic parameters after SPKT and KTA were evaluated.

### Patient and methods

#### Study design

Medical data from patients with IDDM who underwent Simultaneous Pancreas Kidney Transplantation (SPKT) or Kidney Transplantation Alone (KTA) at the University Hospital of Leipzig between 2000 and 2013 were retrospectively analyzed. Our data source comprised a prospectively collected electronical data base. Approval for this analysis was granted by the local ethics committee [AZ: Nr: 111–16-14,032,016]. Patients undergoing re-transplants, living donor kidney transplant, younger than 18 years, and those with missing data were excluded from the study.

#### Outcome measures

Special emphasis was placed on patient and graft characteristics, cardiovascular risk factors, metabolic outcomes and status of PVD. Characteristics included age, gender and body mass index (BMI, weight in kg/ height in m^2^), duration of insulin dependent diabetes mellitus, duration of dialysis, smoking habits, time on the waiting list. Laboratory parameters were HbA1c, low-density lipoprotein (LDL)- cholesterol, high density lipoprotein (HDL) -cholesterol, triglyceride and total cholesterol. Cardiovascular disease and risk factors included information about peripheral arterial obstructive disease (PAD), coronary heart disease (coronary artery bypass graft (CABG)/ stent), blood pressure values and amount of antihypertensive medication. Peri- and post-transplant data included information on immunosuppressive therapy as well as patient and organ graft function.

### Peripheral vascular complications (PVC)

Progression of PVD was determined by evaluating the number of patients with PVCs, defined as any midfoot and limb amputation, ischemic ulceration, lower extremity bypass surgery or angioplasty.

For the analysis of PVCs, the type of radiologic examinations (ultrasonography, conventional angiography, computed-angiography (CTA), magnetic resonance- angiography (MRA)), the type of PAD presentation (Claudicatio, chronical limb ischemia, Ulceration/ gangrene, **Fontaine-classification**), the level of PAD (stenosis, occlusion, micro-arterial disease) as well as the treatment modalities (endovascular treatment, bypass surgery, amputations) were analyzed pre- and post-transplant.

During follow-up the number of amputations, as well as amputation time after transplantation and type of amputations were recorded and analyzed. A major amputation was defined as running through, or being proximal to, the tarsometatsarsal joint whereas a minor amputation was defined as being distal to the tarsometatarsal joint.

#### Organ procurement and transplantation

Pancreas and kidney grafts were procured following the international standards and guidelines provided by the Deutsche Stiftung für Organtransplantation (DSO) [13, 14], as described previously. In short, the pancreas was explanted in a no-touch technique en-bloc with the spleen and duodenum. Back table preparation included removal of the spleen and peripancreatic fat. Reconstruction of the superior mesenteric and the lineal artery was performed using the donor iliac Y-graft.

The pancreas was transplanted into the right iliac fossa using a standard technique with an intraperitoneal location in the right iliac fossa. The Y-graft was anastomosed to the recipient’s common iliac artery using 6–0 Prolene running sutures. The portal vein was connected to the inferior vena cava of the recipient [15]. Exocrine drainage was carried out with a hand-sutured side-to-side duodenojejunostomy 40 cm beyond the flexure of Treitz [15, 16]. All kidneys were transplanted into the contralateral iliacal fossa. Vascular anastomoses were performed to the external iliac artery and vein. The ureter was implanted into the bladder according to the Lich-Gregoir technique using a double J catheter as an intraureteral splint [17]. Splint removal was performed 3–4 weeks after successfull transplantation.

Kidneys in in the KTA group were implanted into the right or left iliac fossa through an extraperitoneal Hockey stick access.

#### Immunosuppression

The immunosuppressive protocol consisted of an induction therapy followed by triple maintenance therapy.

For induction, antithymocyte globulin (ATG, Thymoglobulin) with a dose of 4 mg/kg body weight was applied as a single dose before transplantation and followed by a 1 mg/kg body weight infusion on postoperative days 1–3. As an alternative, the interleukin-2 receptor antagonist basiliximab (Simulect®, 20 mg before transplantation and 20 mg on post-operative day 4) was used in patients with contraindications for ATG therapy. Main contraindications comprised Leukocytopenia, Thrombocytopenia or prevalent urinary tract infections.

Maintenance therapy included calcineurin inhibitors (Cyclosporin (Sandimmun Neoral® or Tacrolimus (Prograf®), and/or antimetabolites (Sirolimus (Rapamune®), Mycofenolate Mofetil (MMF); (Cell Cept®, Myfortic®) and tapered steroids (Prednisolone®). Whole blood levels of Tacrolimus were adjusted to 10–12 ng/ml for the first 3 months and 8-10 ng/ml for month 4 to 12. One year after transplantation levels were reduced to 6 to 8 ng/ml. In parallel, MMF was given at an oral dose of 1 g (Cell Cept®) or 720 mg (Myfortic®) twice daily.

A rapid steroid-tapering regimen was applied in all our patients, starting with 500 mg methylprednisolone intraoperatively to reach a dose of 25 mg prednisolone at the end of the first week after transplantation. Further reduction intended a daily maintenance dose of 5 mg. Whenever possible, steroids were rapidly withdrawn and discontinued at the end of the first year.

#### Statistical analysis

SPSS software, version 21.0 (SPSS Inc., Chicago, Illinois, USA) and Graphpad Prism software, version 8.30 (GraphPad Software Inc., La Jolla, CA) were used for statistical analysis and graphs. All values are presented as mean ± standard deviation (SD). Baseline data were compared with appropriate statistical significance test including Student’s t–test, χ2, analysis of variance (ANOVA), Kruskal-Wallis and Wilcoxon–Mann–Whitney test. *P* values < 0.05 were considered significant. Survival rates were calculated according to Kaplan Meier, and log rank test was used to test for significance.

Primary endpoint was graft survival. Graft survival was calculated as the time from initial transplant to graft failure, re-transplant, or all-cause death. If a recipient was alive or lost to follow-up at time of last contact, then survival time was censored at time of last contact. Secondary end-point was occurrence of PVC, defined as any midfoot and limb amputation, ischemic ulceration, lower extremity bypass surgery or angioplasty occurring post-transplant.

Analysis were performed for the entire patient sample (category 1), and adjusted subgroups (category 2), consisting of all patients with preoperative PAD and/or CHD, cardiovascular risk factors: metabolic syndrome (here defined as: systolic blood pressure > 140 mmHG, Triglyceride-levels > 1.7 mmol /L, recipient BMI > 25 kg/ m^2^) and recipient age > 45 years.

Cox proportional hazards regression models for multivariate analyses were used to estimate via hazard ratio (HR) the effect of transplantation (SPKT versus KTA) on primary and secondary events after adjusting for the above described risk factors and categories.

## Results

### Baseline characteristics

Overall study population included 127 patients receiving a Simultaneous Pancreas Kidney transplantation (SPKT, *n* = 101) or Kidney Transplantation Alone (KTA, *n* = 26). Mean follow-up period was 101 ± 34.4 months. Donor, recipient, and pre-transplant baseline characteristics according to transplant types are summarized in Table 1. In the KTA group, 9 (34%) patients had type 1 and 17 (66%) patients had type 2 insulin dependent diabetes mellitus.

**Table 1:** Characteristics of the overall study population of donors, recipients before transplant, and immunosuppressant medication for Simultaneous Pancreas Kidney transplantation (SPKT) and Kidney Transplantation Alone (KTA) (category 1). Data are shown as mean ± SD. BMI, body mass index; ALT, anti-lymphocyte globulin; ATG, anti-thymocyte globulin; IL-2 RA, Interleukin-2 receptor antagonist; CNI, calcineurin inhibitor; AP drug, antimetabolite; MMF, Mycofenolate mofetil; SRL, sirolimus

Variables	SPK (*n* = 101)	KTA (*n* = 26)	*P*-value
Donor			
Age, years	24.2 ± 11.9	59.7 ± 17.4	< 0.001
Gender			
Male	60 (69.4%)	12 (46.2%)	0.224
Female	41 (40.6%)	14 (53.8%)	
BMI, kg/m2	22.4 ± 3.1	25.4 ± 3.5	< 0.001
Recipient			
Age, years	42.9 ± 8.8	61.5 ± 8.6	< 0.001
Gender			
Male	57 (56.4%)	21 (80.8%)	0.023
Female	44 (43.6%)	5 (19.2%)	
BMI, kg/m^2^	25.1 ± 4.2	28.6 ± 3.1	< 0.001
Smokers, n	21 (21%)	7 (27%)	0.501
Duration of IDDM, years	26.6 ± 8.5	18.9 ± 8.9	< 0.001
Duration of dialysis, years	2.7 ± 2.6	7.4 ± 4.1	< 0.001
Hypertension			
Yes	87 (86.1%)	23 (88.5%)	0.756
No	14 (13.9%)	3 (11.5%)	
Blood pressure, mmHg			
Systolic	135 ± 17	141 ± 18	0.03
Diastolic	76 ± 10	82 ± 11	0.02
Antihypertensive drugs, n	2.6 ± 1.3	2.0 ± 1.5	0.046
Arterial obstructive disease			
Yes	17 (16.8%)	8 (30%)	0.114
No	84 (83.2%)	18 (70%)	
Coronary heart disease			
Yes	29 (28.7%)	19 (73.1%)	< 0.001
No	71 (71.3%)	7 (26.9%)	
Time on waiting list, months	10.4 ± 13.1	22.3 ± 28.4	0.012
Pre-emptive transplant	22 (24.7%)	2 (7.7%)	0.032
Immunosuppression			
Induction therapy			
ALG/ATG	74 (73.3%)	4 (15.4%)	0.001
IL2-RA	19 (18.8%)	12 (46.2%)	
None	8 (7.9%)	10 (38.5%)	
CNI			
Tacrolimus	97 (96.0%)	25 (96.2%)	0.979
Cyclosporin	4 (4%)	1 (4.5%)	
AP drug			
MMF	83 (82.2%)	22 (84.6%)	0.002
SRL	14 (13.9%)	0	
Multiple	3 (3.0%)	0	
None	1 (1%)	4 (15.4%)	

To improve comparability of both study populations, subgroups of patient with cardiovascular risk factors and diseases prior to transplantation were analysed (Table 2).

**Table 2:** Characteristics of donors, recipients before transplant, and immunosuppressant medication for Simultaneous Pancreas Kidney transplantation (SPKT) and Kidney Transplantation Alone (KTA) of patients with preoperative cardiovascular diseases and risk factors (category 2). Data are shown as mean ± SD. BMI, body mass index; ALT, anti-lymphocyte globulin; ATG, anti-thymocyte globulin; IL-2 RA, Interleukin-2 receptor antagonist; CNI, calcineurin inhibitor; AP drug, antimetabolite; MMF, Mycofenolate mofetil; SRL, sirolimus

Variables	SPK (*n* = 22)	KTA (*n* = 20)	*P*-value
Donor			
Age, years	50.5 ± 4.5	61.7 ± 6.7	< 0.01
Gender			
Male	16 (72.7%)	16 (80%)	0.580
Female	6 (27.3%)	4 (20%)	
BMI, kg/m2	27.8 ± 3.9	27.7 ± 2.7	0.884
Recipient			
Age, years	30.7 ± 12.7	60.4 ± 15.8	< 0.01
Gender			
Male	12 (54.5%)	8 (40%)	0.346
Female	10 (45.5%)	12 (60%)	
BMI, kg/m^2^	23.3 ± 2.7	24.8 ± 1.8	0.079
Duration of IDDM, years	27.8 ± 7.9	19.2 ± 9.5	< 0.01
Duration of dialysis, months	3.8 ± 3.1	5.7 ± 1.9	0.05
Blood pressure, mmHg			
Systolic	145 ± 10	148 ± 5	0.07
Diastolic	86 ± 8	91 ± 5	0.05
Antihypertensive drugs, n	3.1 ± 1.2	2.6 ± 1.6	0.237
Arterial obstructive disease			
Yes	17 (77%)	8 (40%)	0.015
No	5 (23%)	12 (60%)	
Coronary heart disease			
Yes	17 (77.3%)	19 (95%)	0.101
No	5 (22.7%)	1 (5%)	
Time on waiting list, months	14.5 ± 16.9	16.1 ± 28.1	0.822
Pre-emptive transplant	9 (41%)	1 (1%)	< 0.01
Immunosuppression			
Induction therapy			
ALG/ATG	15 (68.2%)	4 (20%)	0.006
IL2-RA	5 (22.7%)	9 (45%)	
None	2 (4.8%)	7 (35%)	
CNI			
Tacrolimus	22 (100%)	19 (95%)	0.288
Cyclosporin	0 (0%)	1 (5%)	
AP drug			
MMF	19 (86.4%)	17 (85%)	0.049
SRL	3 (13.6%)	0	
Multiple	0	0	
None	0	3 (15%)	

### Peripheral vascular diseases and complications before transplantation

In addition to physical examination, vascular status was evaluated in all patients by imaging. Vascular imaging included 115 duplex sonography examinations, 114 Magnetic Resonance Angiographies (MRA) or Computed Tomography Angiographies (CTA) and 28 conventional contrast angiograms.

Before transplantation, the incidence of Peripheral Vascular Diseases (PAD) and Complications (PVC) of the lower extremity were comparable in both groups (Table 3). Overall, there were 22 PVCs (22%) in the SPKT group and ten PVCs (38%) in the KTA group before transplantation (*P* = 0.10). In total, 17 patients (17%) in the SPKT group were diagnosed with PAD before transplantation compared to eight patients (30%) in the KTA group (*P* = 0.11). In the SPK group, eleven patients were diagnosed with superficial femoral artery (SFA) lesions (occlusion or stenosis), four patients suffered from tibial lesions and two patients had popliteal lesions prior to transplantation. In the KTA group, three patients were diagnosed with SFA lesions, three patients with tibial lesions and two with popliteal lesions or a combination.

**Table 3:** Peripheral Vascular Diseases (PAD) and Peripheral Vascular Complications (PVC) after Simultaneous Pancreas Kidney Transplantation (SPKT) and Kidney Transplantation Alone (KTA)

Variables	Before transplantation			After transplantation		
	SPKT (*n* = 101)	KTA (*n* = 26)	*P*-value	SPKT (*n* = 101)	KTA (*n* = 26)	*P*-value
Peripheral vascular diseases						
Fontaine-Classification						
I	84 (83.2%)	18 (69.2%)	0.11	80 (79.2%)	15 (57.7%)	0.02
II a	8 (7.9%)	4 (15.4%)	0.24	5 (5%)	3 (11.5%)	0.21
II b	4 (4%)	1 (3.8%)	0.97	7 (6.9%)	2 (7.7%)	0.89
III	1 (1%)	1 (3.8%)	0.29	3 (3%)	2 (7.7%)	0.26
IV	4 (4%)	2 (7.7%)	0.42	6 (5.9%)	4 (15.4%)	0.11
Peripheral vascular complications						
Number of patients	17 (17%)	8 (31%)	0.11	21 (21%)	11 (42%)	0.02
Ischemic Ulceration	5 (5%)	2 (7.7%)	0.58	6 (6%)	4 (15%)	0.11
Revascularization	9 (9%)	4 (15%)	0.33	14 (14%)	8 (31%)	0.04
Amputation	8 (8%)	5 (19%)	0.08	12 (12%)	6 (24%)	0.05
Total PVC	22 (22%)	10 (38%)	0.10	32 (32%)	18 (69%)	< 0.001

Before transplantation, revascularization procedures included six PTAs and three bypass surgeries in the SPK group. Six patients underwent amputations and in two cases a second amputation was necessary. This resulted in six minor and two major amputations. In the KTA group, two PTAs and two bypass surgeries were performed. And four minor, and one major amputation (2 repeats) were necessary in four KTA patients.

### Peripheral vascular diseases and complications after transplantation

**Fig. 1:**
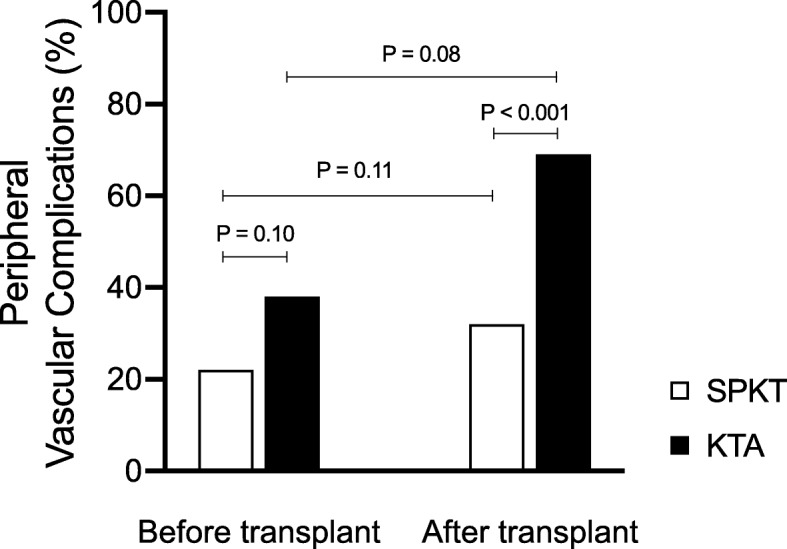
Incidence of peripheral vascular complications before and after Simultaneous Pancreas Kidney Transplantation (SPKT) and Kidney Transplantation Alone (KTA)

Number of PVCs after transplantation are shown in Table 3. There was a significant increase in PVCs in patients received KTA when compared to patients after SPKT (*P* < 0.001) (Fig. 1).

Of 21 patients who developed ischemic symptoms after SPKT, 15 presented with claudicatio, and six established foot ulcers or toe gangrenes. Clinical symptoms of ischemia occurred at a mean time of 27 ± 9 months in SPKT group and 18 ± 9 months in the KTA-group (*P* = 0.18).

In four patients of the SPKT group who had PVC before transplantation, no PVC occured after transplantation. In the KTA group, 13 patients had no PVCs and two patients with PVC before transplantation developed no new PVC.

In the SPKT group, revascularization included four PTAs, two operative thrombendarterectomies (TEA) and eight bypass procedures. Five of these procedures failed, leading to amputation in one patient. Eight revascularization procedures were performed in the KTA group, including two PTAs, one operative TEA and five bypass surgeries. Three of these procedures failed, resulting in limb amputations in all patients.

In total, twelve amputations (8 minor, 4 major) were performed in nine SPKT recipients (9%). The most frequent form of amputation was toe amputations (*n* = 5), followed by transmetatarsal (*n* = 3) and below-knee amputation (*n* = 3) and thigh amputation (*n* = 1). In contrast, six amputations (5 minor, 1 major) were done in five patients of the KTA group (19%). Again, the most common form of amputation was toe amputations (*n* = 4), followed by transmetatarsal (*n* = 1) and below-knee amputations (*n* = 1). Amputations were performed 32 ± 6 months after transplantation in the SPKT group and 22 ± 4 months after transplantation in the KTA group (*P* = 0.284).

Multivariate analysis adjusted for risk factors in category 2 showed that patients undergoing KTA were more likely to have secondary events (HR 2.8; 95% CI: 1.1–8.0; *P* = 0.047).

### Side of peripheral vascular disease and transplant location

There were 21 ischemic events of the lower extremity in the SPKT group. Four of these were on the right side, distal to where the pancreas graft was implanted and 17 on the left side where the kidney was implanted. Using logistic regression analysis, clinical symptoms of ischemia were more likely attributed to the left lower extremity (OR 3.58; 95%CI: 1.1–11.3; *P* = 0.032).

In the KTA group, eleven ischemic events were recorded after transplantation. Out of these, seven were located in the right and five were located in the left lower extremity. Logistic regression analysis revealed no significant differences in terms of implantation site of the kidney and development of clinical symptoms of ischemia (*P* = 0.056).

### Metabolic outcome

As shown in Table 4, significant differences of HbA1c levels were detected between both groups (*P* < 0.01). Triglyceride level in patients with SPKT were lower at 3 and 5 years after transplantation when compared to patient with KTA (*P* < 0.01), whereas 5 years after transplantation HDL-levels were significant higher in patients with SPKT compared to patients with KTA (*P* = 0.01). No significant differences were observed in cholesterol, LDL-, and serum creatinine levels between both groups.

**Table 4:** Metabolic outcome during the first 5 years after transplantation. Data are shown as mean ± SD. KTA, kidney transplant alone; SPK, Simultaneous Pancreas Kidney transplantation; POY, postoperative year

Variable	Group		Postoperative year			*P*- value preop vs 5-POY	*P*-value; SPK vs KTA, 5-POY
		Preoperative	1	3	5		
HbA1c, %	SPK	7.8 ± 1.7	5.8 ± 1.2	5.5 ± 1.7	5.6 ± 0.9	< 0.01	< 0.01
	KTA	6.7 ± 0.9	6.4 ± 1.3	7.1 ± 2.1	7.8 ± 1.9	< 0.01	
Cholesterol, mmol/l	SPK	5.3 ± 2.1	4.9 ± 1.1	4.6 ± 0.9	4.9 ± 0.8	0.07	0.99
	KTA	4.9 ± 1.6	5.3 ± 1.8	5.1 ± 1.2	5.0 ± 1.4	0.76	
Triglyceride, mmol/l	SPK	2.1 ± 1.2	1.6 ± 1.1	1.3 ± 1.0	1.4 ± 0.9	< 0.01	< 0.01
	KTA	2.6 ± 1.2	2.9 ± 1.7	2.2 ± 1.3	3.1 ± 1.5	0.08	
LDL- cholesterol, mmol/l	SPK	2.6 ± 1.4	2.7 ± 0.6	2.6 ± 1.1	2.5 ± 0.8	0.82	0.84
	KTA	2.8 ± 1.4	3.1 ± 1.2	2.8 ± 0.9	2.7 ± 0.8	0.84	
HDL- cholesterol, mmol/l	SPK	1.5 ± 0.6	1.4 ± 0.3	1.7 ± 0.5	1.6 ± 0.3	0.89	0.01
	KTA	1.2 ± 0.4	1.1 ± 0.5	1.2 ± 0.2	1.3 ± 0.3	0.67	
Serum creatinine, μmol/l	SPK	156 ± 21	127 ± 8	132 ± 10	135 ± 11	0.04	0.12
	KTA	210 ± 29	161 ± 15	178 ± 25	195 ± 37	0.09	

### Patient and organ survival

**Fig. 2:**
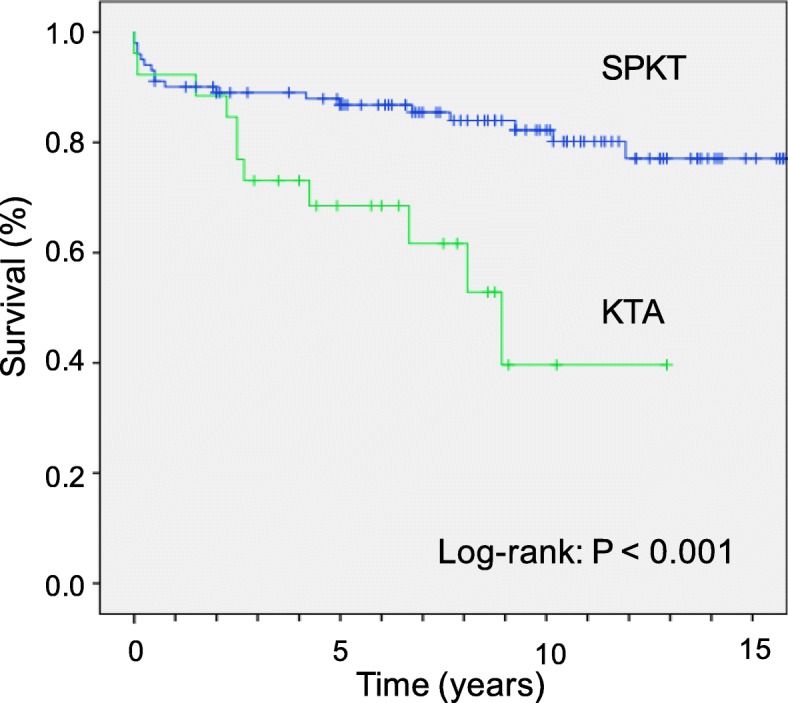
Patient survival after Simultaneous Pancreas Kidney Transplantation (SPKT) and Kidney Transplantation Alone (KTA) in the Subgroup Category 1

The 1-, 3-, and 5- and 10-year overall survival rates in patient after SPKT were 92, 89, 88 and 82%, respectively, and 92, 73, 69 and 40% after KTA (*P* < 0.001; Fig. 2). The 1-, 3-, and 5- and 10-year pancreas graft survival rates were 84, 80, 79 and 75%, respectively. Kidney graft survival rates were 89, 85, 80 and 69%, for the SPKT group, and 81, 65, 58 and 26% for the KTA group, respectively (*P* < 0.001).

**Fig. 3:**
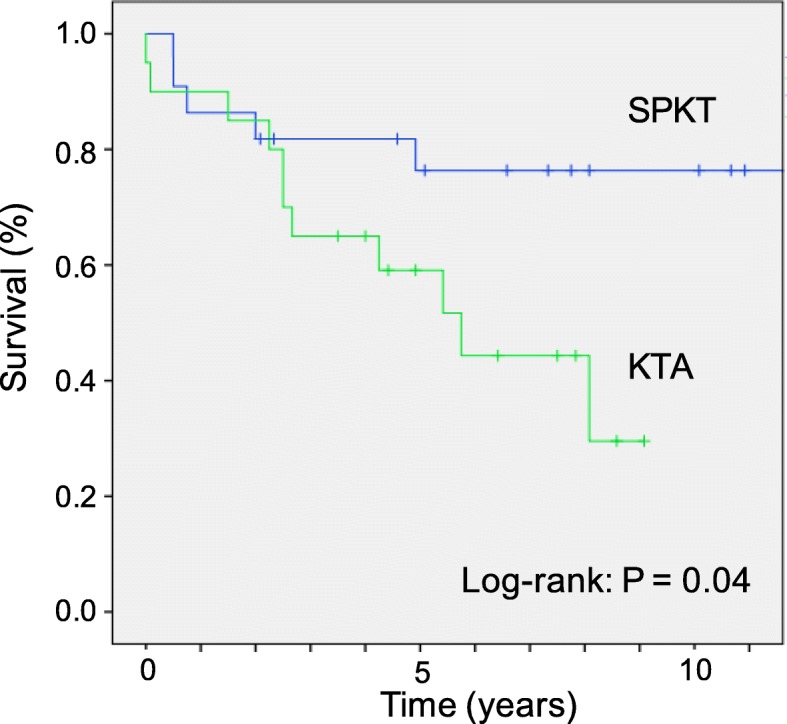
Patient survival after Simultaneous Pancreas Kidney Transplantation (SPKT) and Kidney Transplantation Alone (KTA) in the Subgroup Category

When SPKT and KTA were examined in relation to patient death in Cox regression models for the entire patient cohort (category 1), patients undergoing KTA were significantly associated with death (KTA versus SPKT; HR 3.16 (95%CI: 1.45–6.82; *P* = 0.003). Regarding kidney graft survival, patients undergoing SPKT were associated with a 68% reduction for kidney graft failure (HR 0.32; 95%CI: 0.17–0.6; *P* < 0.001). In contrast, among patients in the risk category 2 after adjustment for multiple confounders in Kaplan-Meier analysis and Cox regression models, patients undergoing KTA were significantly associated with death (KTA versus SPKT; HR 2.91 (95%CI: 0.99–8.47); *P* = 0.05; Fig. 3).

Analyzing SPKT and KTA in relation to kidney graft survival in this category, patients undergoing KTA had greater incidence of graft failure compared to SPKT recipients (HR 3.58; 95%CI: 1.23–10.4; *P* = 0.019).

## Discussion

Our study highlights some important findings. Peripheral vascular disease is a serious affection in patients qualifying for kidney and/or combined pancreas kidney transplantation. From a demographic perspective, patients evaluated for KTA were significantly older and had a higher number of cardiovascular risk factors including higher BMI, high blood pressure and presence of cardiovascular disease making a direct comparison with younger and leaner IDDM1 patients not feasible. Risk factor adjusted multivariate subgroup analysis, however, revealed that patients undergoing KTA are more likely to have progression of PVD leading to increased rates of PVC. A functioning pancreas and kidney allograft in SPKT patients with its accompanying metabolic benefits seems to significantly reduce progression of PVD and led to decreased rates of PVC in IDDM1 patients. Mean follow up of 8.4 years (101 ± 34 month) revealed a significant increase of perivascular complications in KTA patients. This increase was paralleled by a significant raise of parameters relating to the patients’ metabolic risk profile like HbA1c and Triglycerides. In contrast, our diabetic patients even displayed a decrease in their metabolic risk profile after SPKT.

In terms of vascular protective effects our data are in line with recently published work which demonstrated a regression of coronary atherosclerosis in IDDM1 patients after successful SPKT [18]. Our data also confirm recent evidence of improved long term survival for SPKT predominantly 5 years after transplantation [19].

Patients with end stage renal disease (ESRD) have an increased risk for arteriosclerosis and PVD [20, 21]. This risk is even higher in IDDM1 patients with ESRD most probably due to the larger amount of concomitant metabolic disorders.

In this context, age, prior cardiovascular events and state of aortic calcification have been shown to be good markers for the identification of patients with higher risks for mortality [22]. Our data additionally show that an altered serologic metabolic risk profile such as high HbA1c and triglyceride levels significantly contribute to increased perivascular complications and amputations.

Peripheral arterial disease affects 3–12% of the global population [23]. The prevalence in individuals with ESRD, IDDM1 who are on hemodialysis (HD) is even higher, ranging from 17 to 48%. The annual incidence of PAD-related amputation within this group has been reported to be 0.012 to 0.05% [24]. The risk of amputation in SPKT patients with PAD ranges from 9.5 to 23% [25]. In our study we investigated amputations in SPKT and KTA patients after a minimum of 5 years follow up and found an incidence rate of 13 and 31% respectively. Similar results were reported by Biesenbach et al. who also found a significant lower incidence of amputations in IDDM1 patients undergoing SPKT compared to KTA (16 and 31%, respectively) [11].

Additional studies have investigated the amputation rate in SPKT and KTA patients, however, data are inconsistent with those reported previously, and the effects of a functioning pancreas allograft on secondary macrovascular complications of diabetes mellitus remain uncertain.

Morissey et al. found that despite excellent transplant outcomes and lower risk factors for PAD, SPKT recipients had a greater incidence of PAD related complications more than 4 years after transplantation [9]. Recent long-term data by MacCraith et al. indicate that also after a 10 year follow up, patients are at significant greater risk of amputation after SPKT compared to KTA [10]. Increased risks of PAD in SPKT recipients might in part depend on the technique of pancreas transplantation. Systemic portal drainage generates a state of ?A3B2 show $132#?>peripheral hyperinsulinemia which has been shown to contribute to arteriosclerosis by stimulating vascular smooth-muscle growth and arterial wall lipid deposition [26]. Portal vascular drainage of the pancreas allograft would lessen the degree of hyperinsulinemia after transplantation, however the effect of this change on the progression of arteriosclerosis and its complications is unknown [27].

Understanding the pathophysiology of diabetes mellitus and its vascular complications is crucial for the development of new treatment strategies to prevent vascular problems. An abnormal metabolic state is commonly linked to long-term immunosuppression. Immunosuppression adversely affects different components of the vasculature leading to inflammation and hypercoagulation, both contributing to neointima proliferation and atherosclerosis [28]. In this context, impaired nitric oxide homeostasis plays a major role in limiting vascular inflammation. We have recently shown that a complex interplay of redox related inflammatory processes have a major impact on smooth muscle cell proliferation and atherosclerosis development in an experimental model of murine aortic transplantation [29].

Modern immunosuppressive agents like tacrolimus and rapamycin have been associated with a reduction in vascular narrowing a central feature for both chronic rejection and atherosclerosis [30]. The immunosuppressive therapy in our SPKT and KTA groups were different. All SPKT recipients received an induction therapy consisting of anti-thymocyte globulin or an IL-2 receptor antagonist followed by a standard triple therapy of cyclosporine or tacrolimus, MMF and tapered steroids. KTA recipients were less likely to receive induction therapy and in case only IL-2 receptor antagonists were applied. Maintenance therapy consisted of cyclosporine, tacrolimus or rapamycin, MMF and tapered steroids. However, the hypothesis that different immunosuppressive regimens in SPKT and KTA patients could be held responsible for the difference in PVD after transplantation is statistically not supported by our data.

Our study has several limitations. The comparison of SPKT with KTA hangs loose, due to the fact that SPKT recipients are younger and more robust. In spite of lack of statically significance in PVD between our SPKT and KTA recipients, by nature the vascular system of our young SPKT recipients has not suffered for the same amount of time from detrimental metabolic conditions when compared to patients in the KTA group. Therefore, the conclusion that superior outcomes for SPKT are related to the pancreas transplant alone or the type of patient receiving the graft must be conceived with caution [19].

## Conclusions

In subgroup analysis, we could demonstrate that at 8.4 years of functioning pancreas graft the progression of macrovascular diseases is significantly reduced in diabetic patients with SPKT when compared to recipients of KTA. This vascular protective effect after SPKT could be explained by a better vascular risk profile of SPKT recipients compared to KTA recipients. Furthermore, our study demonstrates that SPKT and KTA can be performed safely in diabetic patients with ERSD even in (a) symptomatic patients with PAD, without deterioration of lower extremity ischemia. Further studies are surely required, in lager series, to investigate the natural history and course of PVD before and after SPKT compared to KTA in diabetic patients.

## Data Availability

Our database contains highly sensible data which may provide insight in clinical and personnel information about our patients and lead to identification of these patients. Therefore, according to organizational restrictions and regulations these data cannot be made publically available. However, the datasets used and/or analyzed during the current study are available from the corresponding author on reasonable request.

## Abbreviations

ADA: American Diabetes Association
ANOVA: Analysis of variance
BMI: Body mass index
CABG: Coronary artery bypass graft
CTA: Computed Tomography Angiography
ERDS: End stage renal disease
ET: Eurotransplant
HDL: High-density lipoprotein
IDDM: Insulin dependent diabetes mellitus
KTA: Kidney Transplantation Alone
LDL: Low-density lipoprotein
MMF: Mycophenolate mofetil
MRA: Magnetic Resonance Angiography
PAD: Peripheral arterial disease
PVC: Peripheral vascular complications
PVD: Peripheral Vascular Disease
SD: Standard deviation
SPKT: Simultaneous pancreas-kidney transplantation
